# Manpower cost for a hypertension health campaign: A cross-sectional study

**DOI:** 10.51866/oa.3l4

**Published:** 2023-11-19

**Authors:** Hooi Chin Beh, Ping Foo Wong, Bee Nah Chew, Yook Chin Chia

**Affiliations:** 1 MBBS FRCP FAFPM, Department of Medical Sciences, School of Medical and Life Sciences, Sunway University, 5 Jalan Universiti Bandar Sunway, Selangor, Malaysia.; 2 Department of Primary Care Medicine, Faculty of Medicine, Universiti Malaya, Kuala Lumpur, Malaysia. Email: ycchia@sunway.edu.my; 3 MBBS, MMed (Family Medicine), Department of Primary Care Medicine, Faculty of Medicine, Universiti Malaya, Kuala Lumpur, Malaysia.; 4 MBBS, Dr Fam Med, MAFP, FRACGP, Cheras Baru Health Clinic, Jalan 16, Kampung Cheras Baru, Off Jalan Kuari, Cheras, Kuala Lumpur, Malaysia.; 5 MBBS, Department of Primary Care Medicine, Universiti Malaya Medical Center, Kuala Lumpur, Malaysia.

**Keywords:** Hypertension, Cost of manpower, Health campaign

## Abstract

**Introduction:**

The overall prevalence of hypertension is high, and many people are unaware of their condition. Screening campaigns can effectively identify this group of patients. The study aimed to determine the cost of manpower for a health campaign for detecting undiagnosed hypertension and the prevalence of hypertension.

**Method:**

This cross-sectional study was conducted at two health centres. Sociodemographic characteristics, hypertension and treatment statuses were recorded. Blood pressure (BP) was measured by either doctors or nurses using automated BP machines. The cost of manpower was calculated as the average salaries of manpower during the 3-day health campaign divided by the total number of days. The final sum was the cost of detecting undiagnosed hypertension.

**Results:**

A total of 2009 participants median age = 50 (IQR = 18-91) were included in the study. The overall prevalence of hypertension was 41.4% (n=832). Among the patients with hypertension, 49.2% (n=409) were unaware of their hypertension status. Conversely, 21.1% (n=423) were known to have hypertension, among whom 97.4% (n=412) were on medications. Among those who were on medications, 49% (n=202) had good BP control. The average total cost of manpower during the 3-day health campaign was RM 5019.80 (USD 1059). The cost of detecting an individual with elevated BP was RM 12.27 (USD 2.59).

**Conclusion:**

The prevalence of hypertension and unawareness is high. However, the average cost of manpower to detect an individual with elevated BP is low. Therefore, regular public health campaigns aiming to detect undiagnosed hypertension are recommended.

## Introduction

The global prevalence of hypertension is high. According to the Global Burden of Disease Study, hypertension is the leading preventable cause of death and the third leading cause of disability.^1^ Most cardiovascular morbidity and mortality are attributed to undiagnosed hypertension.^2^ In developing countries, 71.8% of individuals are unaware that they have elevated blood pressure (BP).^3^ Despite the availability of efficacious therapeutic agents, many patients with elevated BP do not take antihypertensive medications. Much of this is attributed to unawareness of the presence of elevated BP.4 The National Health and Morbidity Survey 2019 conducted in Malaysia showed that the overall prevalence of hypertension among adults aged >18 years was 30%, while that of unawareness of the condition was 47%.^4^

In 2022, the World Health Organization’s Global HEARTS Initiative and the HEARTS in the Americas Initiative were implemented to catalyse the application of the latest guidelines in prioritising the prevention and control of hypertension to improve patients’ well-being and reduce cardiovascular complications.^5^ Prevention is better than cure, but this comes with a cost. The costs associated with healthcare continue to be a major concern worldwide. In Malaysia, the primary healthcare treatment or first-line treatment in public healthcare settings, including blood tests, bio-imaging and medications, incurs a fee ranging from RM 1 to RM 5 per visit. Conversely, the costs of accessing similar services at private clinics/hospitals are regulated by the Private Healthcare Facilities and Services Amendment Order 2013.^6^ Initial general and specialist consultations cost RM 30–125 and RM 80–235, respectively.^6^ Such costs could be financially distressing to certain groups of the population, especially those with a lower socioeconomic status. In Malaysia, there are many strategies and recommendations that can be applied to overcome this problem. One of them includes a general health campaign that aims to provide free screening and counselling to the public and the community. The Health Care Scheme for the B40 group (bottom 40 according to the Malaysian household income classification) is a government initiative implemented by the Ministry of Health that aims to sustain the healthcare needs of low-income groups by focusing on screening of non-communicable diseases.^7^

The present study aimed to determine the prevalence of unawareness of elevated BP and calculate the cost of manpower for detecting undiagnosed hypertension. Further, the prevalence of known hypertension, status of antihypertensive medication or treatment and hypertension control were evaluated.

## Methods

In this cross-sectional study, a hypertension health campaign was conducted in conjunction with World Hypertension Day at two separate centres (one teaching hospital and one public health clinic). The campaign was conducted for 3 days. Individuals aged ≥18 years who were visiting the health facilities were included, while those who were pregnant were excluded. Verbal consent was obtained. Thereafter, the participants were asked a few simple questions regarding their sociodemographic data and hypertension and treatment statuses.

The participants’ BP was measured by either doctors or nurses using automated digital BP devices in accordance with the Malaysian Clinical Practice Guidelines (CPGs) on the Management of Hypertension.^8^ The participants were seated for 1 min before commencement of the BP measurements. The BP was measured twice with a 1-min interval, and the average of the two readings was calculated. We used a standard bladder that encircled at least 80% of the arm circumference, with the width encircling at least 40%. The cuff was placed at the heart level. Hypertension was defined as a BP of ≥140/90 mmHg.^9^ Hypertension was considered controlled when the BP was <140/90 mmHg, as recommended in the CPGs on the Management of Hypertension.^8^

The participants who had elevated BP were directed to doctors’ station where their BP was measured again using a mercury sphygmomanometer via the above-mentioned method. With the auscultatory method, we used phase I and V (disappearance) Korotkoff sounds to identify systolic and diastolic BP, respectively. The BP reading with the higher value was used as the reference.

All participants were given information on healthy lifestyles regardless of their BP readings. The participants who were found to have elevated BP were counselled and given specific instructions to consult their family doctor or return to our clinic for confirmation of their hypertension status. We also provided them with a small advice paper with their BP reading to ease the process of follow-up.

## Cost calculation

The cost of manpower was calculated as the sum of the average salary of a doctor per month divided by 22 working days multiplied by four doctors multiplied by 3 days and average salary of a nurse per month divided by 22 working days multiplied by four nurses multiplied by 3 days. The total sum of both salaries was divided by 409 individuals who were not known to have hypertension but had high BP. The detail of the formula was shown in table 3.

## Statistical analysis

Data were statistically analysed using the Statistical Package for the Social Sciences (version 23). Continuous data that were normally distributed such as systolic BP and diastolic BP were described as means and standard deviations while age was described as median and range as it was not normally distributed. Categorical data, including independent variables such as sex, race, hypertension status, treatment status and hypertension control, were reported as frequencies (percentages). All data were fully presented without restriction.

## Results

A total of 2009 participants were included in the study. The median age of the participants was 50 with interquartile range (IQR) = 1891 years. Half were women (50.5%, n=1015). The majority of the participants were Malay (56.3%, n=1132), followed by Chinese (24%, n=483), Indian (16.8%, n=337) and others (2.8, n=57). The prevalence of known hypertension was 21.1% (n=423) (Table 1).

**Table 1:** Demographic profile of the participants (N=2009).

Variable	Value
Age (year), median (IQR)	50 (18-91)
SBP (mmHg), mean ± SD	129.99+17.8
DBP (mmHg), mean ± SD	75.95+11.1
**Sex, n (%)**	
Male	994 (49.5)
Female	1015 (50.5)
**Race, n (%)**	
Malay	1132 (56.3)
Chinese	483 (24)
Indian	337 (16.8)
Others	57 (2.8)
**Hypertension, n (%)**	
No	1586 (78.9)
Yes	423 (21.1)

SBP, systolic blood pressure; DBP, diastolic blood pressure; SD, standard deviation; IQR, interquartile range

The total prevalence of hypertension was 41.4% (n=832). Among the participants with hypertension, the total prevalence of unawareness of the condition was 49.2% (n=409). Among those who were known to have hypertension, 97.4% (n=4l2) were on antihypertensive medications. Among those who were on antihypertensive medications, 49% (n=202) had good BP control (Table 2).

**Table 2:** Awareness and status of treatment and control among the adults with hypertension.

Variable	Frequency (n, %)
**Patients who are known to have hypertension (n=423)**	
On antihypertensive medications	412 (97.4)
Not on antihypertensive medications	11 (2.6)
**Patients who are on antihypertensive medications (n=4l2)**	
Controlled BP	202 (49)
Uncontrolled BP	210 (51)
**Total prevalence of hypertension (n=832)**	
Unaware of their hypertension status^*^	409 (49.2)
Aware of their hypertension status	423 (50.8)

BP, blood pressure

* Unaware of their hypertension status=total prevalence of unawareness

The average salary of a doctor was RM 5302.98, while that of a nurse was RM 3900. The average salary was divided by the number of working days (22 working days in a month, excluding four weekends). The total salary of four doctors and four nurses for the 3-day health campaign was RM 5019.80 (RM 2892.53 for the doctors and RM 2127.27 for the nurses). The number of participants who were unaware of having elevated BP was 409. Accordingly, only RM 12.27 (USD 2.59) was calculated to be required to detect one person who was unaware of having elevated BP (Table 3).

**Table 3:** Cost calculation for detecting an individual who was unaware of having elevated BP.

Variable	Amount (RM)
Average salary of a doctor per month	5302.98
Average salary of a nurse per month	3900
Average salary of a doctor per working day^e^ × four doctors × 3 days	2892.53
Average salary of a nurse per working day^e^ × four nurses × 3 days	2127.27
**Subtotal salary** ^f^	5019.80
**Cost of detecting an individual who was unaware of having elevated BP** ^g^	12.27

BP, blood pressure

e Working day: 22 days in a month (excluding four weekends)

f Subtotal average salary: Average salary of four doctors and four nurses for the 3-day health campaign

g Manpower cost of a health campaign for detecting an individual who was unaware of having hypertension (n=409, 49.2%)

## Discussion

Hypertension is a well-known risk factor for cardiovascular and renovascular diseases.^10-13^ Efforts to reduce this major risk factor are hampered by the high prevalence of unawareness, and hence, opportunities to treat and reduce the BP and cardiovascular risk are missed. Our study showed that the prevalence of unawareness of the presence of hypertension was 49.2%, compared with 47% reported in the National Health and Morbidity Survey 2019.^3^

A few studies have shown that younger adults and men are more likely to be unaware that their BP is elevated.^14-15^ As the prevalence of unawareness of elevated BP is high, many health authorities have invested in public health education and campaigns to raise awareness. Apart from these educational efforts, adopting a different strategy such as actively and regularly organising health campaigns is recommended. However, such an approach would require an increase in overall cost.

In our study, we found that the cost of manpower for detecting an individual who was unaware of having elevated BP was relatively low at RM 12.27 (USD 2.59). In view of such low cost, it is worthwhile to organise regular health campaigns to increase the level of awareness up to 80% as reported in some developed high-income countries such as the USA^16^ and some Asian countries.^17^ If the level of awareness is increased, individuals with elevated BP can receive appropriate management as early as possible.

The advantages of health campaigns include counselling individuals on their BP status and providing advice on the benefits of healthy lifestyle changes, which could be achieved simultaneously without incurring additional costs. Studies have also shown that the advices given by doctors at the time of diagnosis have the greatest impact on behavioural changes.^18^ For example, better BP control can be achieved if patients have known cases of diabetes and chronic kidney diseases.^19^ Modifiable risk factors such as higher body mass index, imbalanced diet especially excessive sodium intake, smoking and excessive alcohol consumption can lead to poor control of hypertension. The Dietary Approaches to Stop Hypertension diet, weight loss, regular exercise, reduction of alcohol intake and smoking cessation, particularly when used in combination, are effective strategies in controlling hypertension.^20-21^ In this study, advices were also given during the health campaign by the attending doctors and nurses with no added cost.

Another benefit of our health campaign was that the patients who were unaware of having elevated BP were advised to have a complete medical checkup to reduce the overall risk of cardiovascular events, while those with normal BP were advised to have an annual BP checkup to detect and treat any abnormal BP changes early. A study conducted in Canada found that older patients reported 3.02 fewer annual hospital admissions for cardiovascular disease in the intervention group than in the no screening group.^22^ A systematic review concluded that it is important to confirm the diagnosis of hypertension by applying re-screening intervals and conducting home pressure monitoring following a screening programme.^22^

This study demonstrates the cost-effectiveness of identifying individuals with elevated BP who are unaware of their condition through public health campaigns for hypertension. It is hoped that this finding will encourage more healthcare centres and providers to organise such health campaigns. Accordingly, more individuals who are not aware of their hypertension status can be identified, and the condition can be treated in its early stage. Subsequently, the cardiovascular risks attributed to undiagnosed or poorly controlled hypertension can be reduced. However, when discussing the overall cost-effectiveness of managing hypertension, factors such as doctors’ hesitancy to initiate treatment for patients who are newly diagnosed with hypertension or those who have poorly controlled BP, patients’ adherence to lifestyle changes and medications and the adequacy of supply of antihypertensive agents in healthcare centres should be taken into consideration. In a previous study, it was shown that a community health screening campaign for hypertension that involved education and referral had achieved an excellent linkage to further care and management.^23^

In summary, health campaigns including screenings can be implemented by health authorities, particularly at healthcare centres where many people frequently visit such places. The additional cost incurred is minimal, as all necessary staff and screening facilities are already readily available and in place. This should make such campaigns highly affordable, even in low-resource countries where the prevalence of elevated BP and unawareness of such is increasing along with changes in lifestyle. Health campaigns should be promoted vigorously through the development and implementation of national action plans provided the country is ready to follow up and treat this group of patients. However, it is essential to consider the potential influx of patients newly diagnosed with hypertension who will require long-term follow-up and treatment in the future. Therefore, a well-structured and planned algorithm must be created and implemented to prevent overwhelming the healthcare system. Government and non-government organisations must also enhance their contributions to hypertension screening or health campaigns, enabling national health authorities to implement various strategies aimed at reducing the prevalence of hypertension and increasing the awareness level and treatment rate among patients with hypertension. Additional studies are necessary to determine the cost-effectiveness of screening or health campaigns in other scenarios. Further cost-effectiveness studies on the cost of emergency admissions and treatments of complications resulting from unawareness of hypertension could help emphasise the need for screening.

## Limitations

The prevalence of hypertension observed in our study might not be generalisable to the whole community, as the health campaign was conducted in selected healthcare centres. The participants were also recruited via convenience sampling: Those who were interested and presented themselves at our campaign were screened. Further, the average salary of a doctor and a nurse may differ according to their grade and position. We also did not include the potential cost of space/rental and electricity, as these are already available in the healthcare centres. A full analysis of cost-effectiveness could be more complex, as other healthcare costs such as those of unscheduled emergency visits, hospitalisations and treatments of complications of uncontrolled hypertension would need to be considered.

## Conclusion

The prevalence of hypertension and unawareness remains high. The cost of manpower for identifying one person who is unaware of having elevated BP is relatively low at only RM 12.27 (USD 2.59). With the high level of unawareness and low cost of detecting an individual with hypertension, it is encouraging to hold health campaigns to detect undiagnosed hypertension in the community. Further studies that would conduct thorough cost-effectiveness analyses based on the BP detection rates and costs attributed to cardiovascular morbidity and mortality are needed.

## Data sharing statement

All data generated and/or analysed during the current study can be accessed upon request to the corresponding author.

How does this paper make a difference in general practice?

- In Klang Valley, there are few health campaigns available, and the response of healthcare centres in organising a health campaign is poor.
- Organising a health campaign is beneficial to patients owing to the low cost.
- Awareness of healthy lifestyles, including the Dietary Approaches to Stop Hypertension diet, can be instilled to the public through a hypertension health campaign.
- The study was able to conduct screening using existing trained staff and infrastructures without incurring additional costs.
